# Factors influencing accumulation of Zn, Cu, and Ca in the tissues of spiny-cheek crayfish (*Faxonius limosus,* Rafinesque, 1817)

**DOI:** 10.1007/s11356-023-25318-9

**Published:** 2023-01-23

**Authors:** Magdalena Stanek, Bogusław Chachaj, Szymon Ł. Różański

**Affiliations:** 1 Department of Animal Physiology and Physiotherapy, Faculty of Animal Breeding and Biology, Bydgoszcz University of Science and Technology, Mazowiecka 28, 85-084 Bydgoszcz, Poland; 2 Department of Animal Biology and Environment, Faculty of Animal Breeding and Biology, Bydgoszcz University of Science and Technology, Mazowiecka 28, 85-084 Bydgoszcz, Poland; 3 Laboratory of Chemical Research and Instrumental Analysis, Faculty of Animal Breeding and Biology, Bydgoszcz University of Science and Technology, Mazowiecka 28, 85-084 Bydgoszcz, Poland

**Keywords:** Calcium, Copper, Zinc, Crayfish tissues, Bioaccumulation, Bottom sediment, Water

## Abstract

Both physicochemical and biological factors affect the degree of metal accumulation in crayfish tissues. The content of metals and correlations between the metal concentrations in different tissues and the total length of crayfish is suitable indicators of contamination of the aquatic environment. The aim of the study was to analyse the effect of age and sex of crayfish on the degree of accumulation of Ca, Cu, and Zn in the muscle and exoskeleton. A total of 100 individuals of the spiny-cheek crayfish (*Faxonius limosus,* Rafinesque, 1817) were caught from Głowińsk reservoir (Poland) in October 2019 using fyke nets. Metal concentrations were determined in freeze-dried samples of the abdominal muscle, exoskeleton, bottom sediment, and water using atomic absorption spectroscopy (AAS). Here, we show that the highest concentrations of Zn were found in the muscle of 4-year-old females, Cu in 3-year-old males, and Ca in 4-year-old males. Sex was a significant factor affecting the content of Ca in the muscle and Zn in the exoskeleton. Age was a significant factor affecting the content of Zn, Cu, and Ca in the muscle and Zn and Cu in the exoskeleton. The bioconcentration factor (BCF) of Zn and Cu in the muscle and exoskeleton of spiny-cheek crayfish was much higher from water than from sediments, unlike Ca. Furthermore, we found significant correlation for muscle between Zn and total length in 3-year-old females and 4-year-old males and between Cu and TL in 3-year-old males. Analysing the recommended daily intake (RDI) for the investigated minerals confirmed that the consumption of 100 g of spiny-cheek crayfish muscle could meet daily requirement for Zn up to 27.5%, for Ca in 12.4%, and over 100% for Cu. The conducted analyses confirmed that the consumption of crayfish meat was safe for the health of potential consumers in terms of the analysed metal content.

## Introduction

Metals entering the environment may accumulate in animal tissues as they travel along the food chain through the diet (biomagnification), leading to high concentrations in organisms at the end of the trophic chain (Balzani et al. 2021). Among metals, there are macro- and microelements essential for animals and humans. Unfortunately, it should be noted that potentially harmless metals, e.g. Zn or Cu, become toxic to the organism when certain concentrations are exceeded (Nędzarek et al. 2020).

It has been proposed that crayfish muscle can be considered by the food industry as an alternative source of raw material (Śmietana et al. 2021). According to these authors, the abdomen and chelae muscles can be a source of minerals, too. As confirmed by numerous studies, crabs, shrimps, crayfishes, and lobsters are a valuable source of macro- and microelements essential for humans, but also of toxic metals in the case of water pollution (Balzani et al. 2022; Barrento et al. 2009; Heidarieh et al. 2013; Nędzarek et al. 2020; Raissy et al. 2011). Yet what about the exoskeleton? Crayfish are frequently suggested as bioindicators for monitoring water conditions in polluted areas (Volpe et al. 2020). Research by Bergey and Weis (2007), Protasowicki et al. (2013), and Mackevičiené (2002) showed that in the case of numerous aquatic crustaceans, i.e. shrimps, crabs, or crayfish, toxic metals tend to accumulate in the exoskeleton. This tissue, therefore, has a very important detoxification function because toxic substances are removed from the animal’s body during the moult. The main component of the shells of crustaceans is chitin (poly-β-(1,4)-N-acetyl-D-glucosamine), a natural biopolymer characterised by high bioactivity, biodegradability, and non-toxicity, as well as good chelating property of metal ions (Bhatnagar and Sillanpää 2009; Jaafarzadeh et al. 2015; de Sousa et al. 2020; Złotko et al. 2021). Therefore, chitosan obtained from chitin is already widely used in medicine in nerve or wound repair and in agriculture as a pesticide, growth stimulator, and fruit preservative, but also in environmental protection in the treatment of wastewater (Zhang et al. 2022). Could the spiny-cheek exoskeleton be a rich source of divalent metals such as Zn, Cu, and Ca?

The degree of accumulation of metals in tissues is influenced by physical and chemical factors, i.e. pH, salinity, and temperature of the environment, exposure time, metal concentration (Anastopoulos et al. 2017; Jaafarzadeh et al. 2015), and biological ones (i.e. tissue type, species, size, body condition, and eating habits) (Balzani et al. 2022; Evans-Illidge 1997; Zhang et al. 2019). The accumulation level of metals in crayfish tissues is a good indicator of contamination of the aquatic environmental (Goretti et al. 2016; Kuklina et al. 2014; Varol and Sünbül 2018). Crayfish can accumulate metals from sediment (Varol and Sünbül 2018), water, or food (Mistri et al. 2020). The research of Keteles and Fleeger (2001) confirms that the tendency to accumulate metals in the exoskeleton of crustaceans is an individual property of the organism and is highly dependent on the type of metal. Therefore, an analysis of age and sex of spiny-cheek crayfish was undertaken.

North American spiny-cheek crayfish (*Faxonius limosus* [Rafinesque 1817]) has been widely introduced in Poland, disrupting the local biodiversity (Krzywosz 2004; Holdich and Black 2007). Due to its tolerance of a wide range of environmental conditions, including chemically contaminated and eutrophicated reservoirs (Buřič et al. 2013) and its immunity to crayfish plague due to the new genotype of pathogen *Aphanomyces astaci*, it poses a threat to native noble crayfish (*Astacus astacus* [Linnaeus 1758]) and mud crayfish (*Astacus leptodactylus* [Eschscholz 1823]) (Śmietana 2000). In accordance with the Regulation of the Minister of Agriculture and Rural Development of November 12, 2001 (Journal of Laws No. 138, item 1559) with the amendment introduced in paragraph 8 (Journal of Laws of 2003, No. 17, item 160), spiny-cheek crayfish should be eradicated and the release of captured individuals is not allowed.

The aim of the study was to analyse the effect of age and sex of crayfish on the degree of accumulation of selected metals (Ca, Cu, and Zn) in the muscle and exoskeleton, to analyse correlations between the metal concentrations in different tissues and the total length of crayfish, to analyse the bioaccumulation coefficient, and to assess the percentage of the analysed metals in the water, sediment, muscle and exoskeleton. Such complex analyses and interactions can improve our understanding of metal absorption, as well as enable more accurate estimates of the amount of bioavailable metals present at a given site. Additionally, the determined concentrations of elements with recommendations for their daily intake were compared.

The research hypothesis states that the age of crayfish, to a greater extent than sex, influences the degree of metal accumulation in crayfish tissues. As spiny-cheek crayfish are benthic crustaceans, the BCF of metals may be higher from sediment than from water.

## Material and methods

### Study site and sampling

**Fig. 1:**
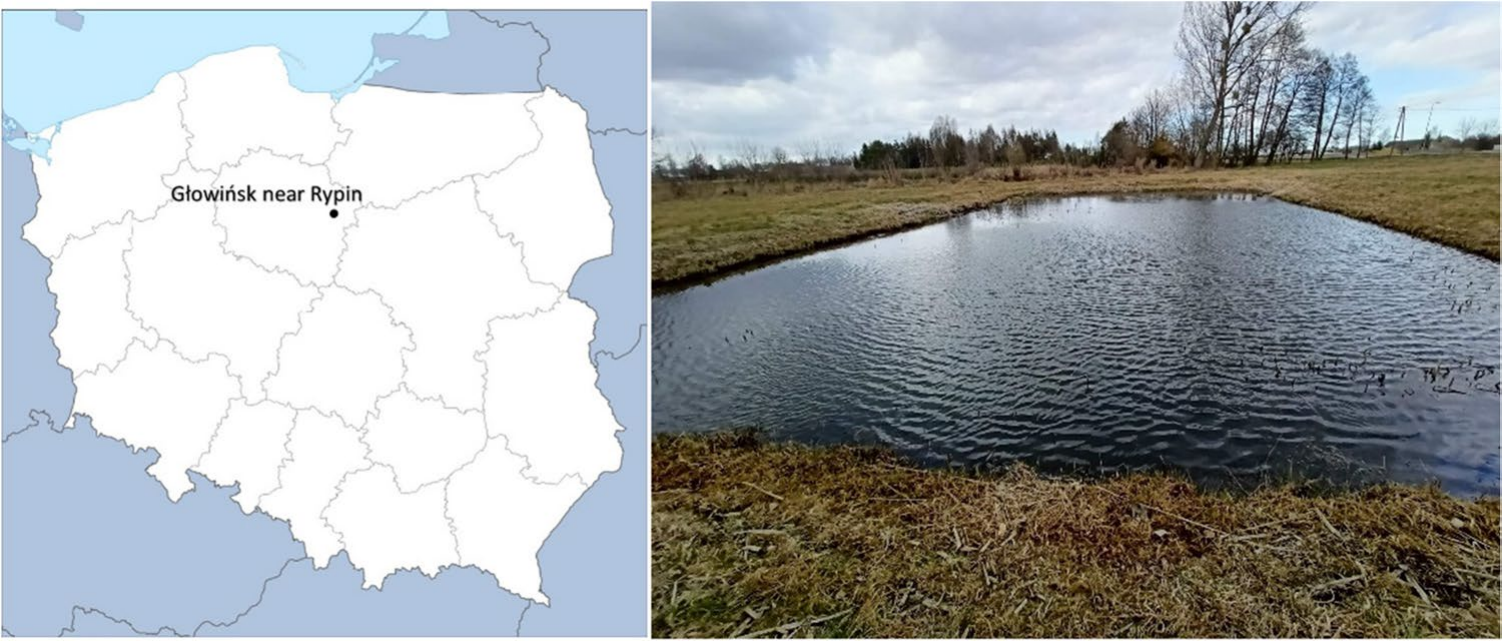
Map of Poland and location of Głowińsk reservoir (Poland)

A total of 50 males and 50 females of the spiny-cheek crayfish were caught in autumn (October 2019) using fyke nets. The traps were placed in the coastal zone of the Głowińsk reservoir near Rypin (north-central Poland) (Fig. 1). Only healthy individuals were included in the study, and crayfish with damaged claws were not considered for the analyses. After washing, the crayfish were placed in water and transported to the laboratory.

The age of the crayfish was determined following the size-age classification of Pieplow (1938). All males occurred as form I (reproductively active form with a well-developed first pair of pleopods) according to the description of Chybowski (2007) and Pielplow (1938). Sexually mature individuals of total length from 80.7 to 104.2 mm (measured from the tip of the rostrum to the end of the telson) have been collected (Table 1). Spiny-cheek crayfish in Polish climatic conditions become sexually mature in the first or second year of life (Holdich and Black 2007; Chybowski 2007) when they reach a total length of 60 mm (Pielplow 1938; Juchno and Chybowski 2003).

**Table 1:** Total length (mm) of 3- and 4-year-old females (♀) and males (♂) of spiny-cheek crayfish (*Faxonius limosus* Raf.) taken from Głowińsk reservoir (Poland)

Individuals		*n*	Min–max (mean)
3-years-old	♀	30	80.7–92.6 (85.88)
	♂	30	80.8–94.2 (88.16)
4-years-old	♀	20	95.2–104.2 (100.80)
	♂	20	95.8–103.9 (99.43)

Water samples were taken from the subsurface layer using water sampling pole from four sampling points (ISO 5667–4:^2016^). One litre of average sample (1000 ml) was filtered using WhatmanTM grade 1 qualitative filter paper and collected in a polyethylene (PE) container. The sample was acidified to pH 1 − 2 with concentrated nitric acid (ISO 5667–3:^2018^). The bottom sediment samples were collected from the same four sampling points using an Ekman-Grab sampler of 2250 ml volume. One litre of average sample (1000 ml) was collected in a polyethylene (PE) container and stored in the refrigerator for analysis (ISO 5667–12:^2017^). Directly after sampling and transportation, the sample was freeze dried (lyophilized) and stored at − 20 °C.

### Metal sample preparation and analysis

For analysis, the abdominal muscle and carapace part of exoskeleton of individuals during inter-moult stage were collected. Crayfish samples of abdominal muscle were preserved in the freezer at − 20 ℃ until further processing. Samples of exoskeleton were air dried. All samples were then freeze-dried in Lyovac GT2 freeze drier by Finn-Aqua (Finland) (parameters: temperature: – 40 °C, pressure: 6·10^−2^ mbar, and duration at least 48 h).

Cu, Zn, and Ca concentrations were determined in freeze-dried samples after aqua regia digestion (ISO 11466:1995) on Thermo Scientific iCE 3000 SERIES spectrophotometer, calibrated using Merck standard solutions (Merck KGaA). The validation of the analysis was conducted on certified standards—certified reference material ERM®-BB422 fish muscle, certified reference material BCR®-670 aquatic plant, and certified reference material CRM027-050 sandy loam 10. The limit of detection (LOD) and the limit of quantification (LOQ) for each element was calculated from the standard deviation of blank samples (S) as LOD = 3 S and LOQ = 10 S (Shehata et al. 2018) (Table 2). The metals in water were determined directly after sampling and transportation. The concentrations of the metals were calculated from linear calibration plots obtained by measurement of the standard solutions. All determinations were made in triplicate and the data for samples of the muscle, exoskeleton and bottom sediments were corrected to oven-dry (105 °C) moisture content (given in mg kg^−1^ dry weight—mg kg^−1^ dw for Cu and Zn and g kg^−1^ dry weight—g kg^−1^ dw for Ca).

**Table 2:** Concentration of metals in certified materials with LOD and LOQ

Element	Certified value (mg kg^−1^)	Determined value (mg kg^−1^)	SD (%)	LOD (mg·l^−1^)	LOQ (mg·l^−1^)
Cu	1.67 ± 0.16^a^ 1.82 ± 0.30^b^ 9.87 ± 0.49^c^	1.53 ± 0.18 1.71 ± 0.29 9.42 ± 0.51	0.18 0.22 0.31	0.0107	0.0356
Zn	16.0 ± 1.1^a^ 24.0 ± 2.1^b^ 51.3 ± 2.63^c^	16.9 ± 1.1 25.4 ± 2.4 52.9 ± 3.23	1.33 1.11 2.48	0.0026	0.0088
Ca	5970 ± 115^c^	5893 ± 197	3.43	0.0169	0.0564

^a^ Certified reference material ERM®-BB422, ^b^ Certified reference material BCR®-670, ^c^ Certified reference material CRM027-050

### Bioconcentration factor

The accumulation of metals in crayfish tissue was measured using BCF. According to Jitar et al. (2015) and Vrhovnik et al. (2013), BCF is defined as follows:

$$\mathrm{BCF }=\mathrm{ Cb}/C$$

where Cb is the concentration of metals in the muscle/exoskeleton; *C* is the concentration of metals in sediments/water.

### Statistical analyses

Statistical calculations were made using Statistica 13.0 software (StatSoft 13.0). Significant differences between the groups were tested with a two-way analysis of variance (ANOVA), and Tukey’s test was used for multiple comparisons. The normality of the data was tested using the Shapiro–Wilk’s test, and the homogeneity of variance was verified by means of the Levene’s test. The level of significance was set at *P* ≤ 0.05. Linear regression analysis was employed to identify relationships between metal concentrations and total length (TL) of spiny-cheek crayfish in the individual tissues for each age and sex group. To assess how well a statistical model predicted an outcome, the coefficient of determination (*R*^2^) was also taken into account, which determines the proportion of variance in the dependent variable that can be explained by the independent variable.

## Results

**Fig. 2:**
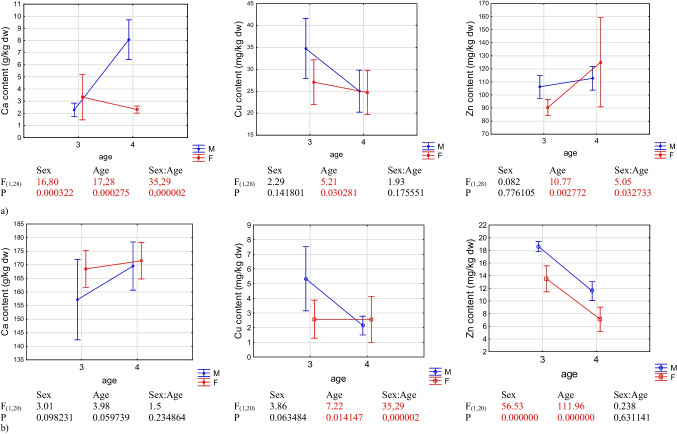
Result of two-way ANOVA for each metal (Ca, Cu, and Zn) and sex in the meat (**a**) and exoskeleton (**b**) of spiny-cheek crayfish (*Faxonius limosus* Raf.) from Głowińsk reservoir (Poland)

Our analyses indicated that metals accumulated in the following sequence: Ca > Zn > Cu in the abdominal muscle, exoskeleton, water, and bottom sediment (Table 3). Figure 2 shows the results of two-way ANOVA for each metal and tissue using both age and sex as predictors, including the values of *F* and *P*. Sex was a significant factor affecting the level of Ca in the muscle and Zn in the exoskeleton. The age of the crayfish was a significant factor affecting the level of Zn, Cu, and Ca in the muscle and Zn and Cu in the exoskeleton, mainly in the male group. The effect of interaction between the analysed variables (age:sex) in the case of Zn and Ca for the muscle and in the case of Cu for the exoskeleton was confirmed (this is shown by the crossed lines on the graphs). For example, the effect of one factor (age) on the dependent variable (Zn) in the muscle changes depending on the effect of the other factor (sex). Significantly high levels of Zn (16.06 mg kg^−1^ dw) and Cu (3.96 mg kg^−1^ dw) were determined in the exoskeleton of 3-year-old individuals. In the case of muscle, significantly high values were found for Zn (117.42 mg kg^−1^ dw) and Ca (5.91 g kg^−1^ dw) in 4-year-old individuals, while the concentration of Cu was nearly 7 mg kg^−1^ dw higher in the tissue of 3-year-old crayfish compared with 4-year-old ones.

**Table 3:** Mean concentration, standard deviation (± SD), minimum and maximum values of metals (Zn, Cu (mg kg^−1^ dw), and Ca (g kg^−1^ dw) in the muscle and exoskeleton of spiny-cheek crayfish (*Faxonius limosus* Raf.), bottom sediment, and water samples taken from Głowińsk reservoir (Poland)

Samples	Zn Mean ± SD Min–max	Cu Mean ± SD Min–max	Ca Mean ± SD Min–max
Muscle	108.88 ± 19.78 83.89–162.75	28.40 ± 8.17 13.50–44.77	4.29 ± 3.01 1.42–11.52
Exoskeleton	12.71 ± 4.45 4.57–19.52	3.16 ± 1.88 0.94–7.60	166.69 ± 10.46 136.69–179.62
Bottom sediment	26.30 ± 5.37 20.41–33.95	12.19 ± 1.77 9.86–14.59	48.04 ± 8.26 40.00–57.40
Water	0.017 ± 0.01 0.015–0.019	0.012 ± 1.77 0.010–0.013	137.28 ± 41.23 112.38–184.88

The highest amounts of Zn were found in the muscle of 4-year-old females, Cu was determined in the highest amounts in 3-year-old males, and the level of Ca was significantly high in the muscle of 4-year-old males (Table 4). Significant differences were found in the concentration of Zn between 3- and 4-year-old females and in the concentration of Cu between 3- and 4-year-old males. Considerably high values of Zn and Cu were found in the exoskeleton of 3-year-old males. A significantly low level of Zn was determined in the tissue of 4-year-old males. Ca concentrations ranged from 157.18 g kg^−1^ dw (3-year-old males) to 171.50 g kg^−1^ dw (4-year-old females), but these values were not significantly different between groups (Table 4).

**Table 4:** Mean concentration of Zn and Cu (mg kg^−1^ dw) and Ca (g kg^−1^ dw) and standard deviation (± SD) in the muscle and exoskeleton of 3- and 4-year-old females (♀) and males (♂) of spiny-cheek crayfish (*Faxonius limosus* Raf.) taken from Głowińsk reservoir (Poland)

Individuals		Zn		Cu		Ca	
		Muscle	Exoskeleton	Muscle	Exoskeleton	Muscle	Exoskeleton
3 +	♀	90.41 ± 5.96^a^	13.52 ± 1.95^a^	27.08 ± 4.85^a,b^	2.58 ± 1.23^a^	3.33 ± 1.78^a^	168.54 ± 6.49
	♂	106.31 ± 12.33^a,b^	18.60 ± 0.77^b^	34.74 ± 9.59^a^	5.34 ± 2.09^b^	2.28 ± 0.78^a^	157.18 ± 14.10
4 +	♀	125.11 ± 32.55^b^	11.58 ± 1.41^a^	24.72 ± 4.79^a,b^	2.58 ± 1.49^a^	2.31 ± 0.28^a^	171.50 ± 6.40
	♂	112.81 ± 12.61^a,b^	7.12 ± 1.81^c^	25.05 ± 6.70^b^	2.15 ± 0.61^a^	8.08 ± 2.29^b^	169.54 ± 8.45

Values in one column marked with different letters, differ statistically significantly at *P* ≤ 0.05 (ANOVA and Tukey post hoc test)

**Fig. 3:**
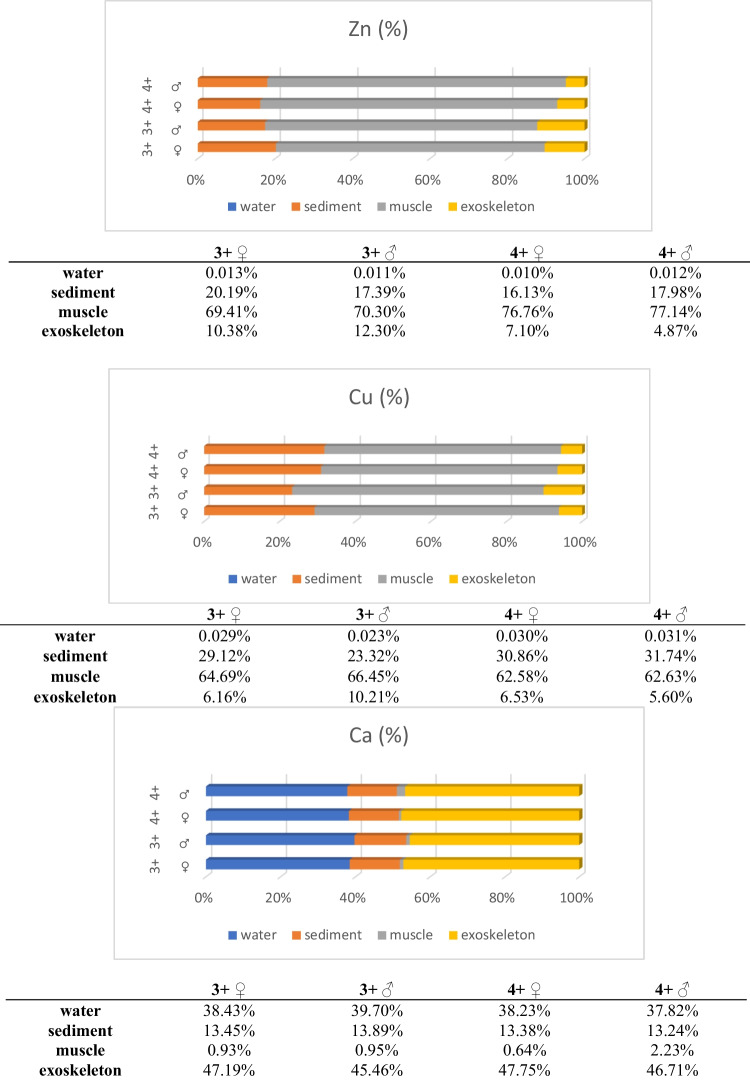
Zn, Cu, and Ca percentage (water/sediment/abdominal muscle/exoskeleton) in 3- and 4-year-old females (♀) and males (♂) of spiny-cheek crayfish (*Faxonius limosus* Raf.) taken from Głowińsk reservoir (Poland)

Figure 3 shows the percentage of the analysed metals in water, sediment, muscle, and exoskeleton. Zn and Cu accumulated in the greatest amounts in the muscle (from 62.60 to 77.17%), and in the smallest amounts in the exoskeleton (from 4.87 to 12.30%). Ca was most abundant in the exoskeleton (77.30% in 4-year-old females to 75.13% in 4-year-old males). In the muscle, the percentage of Ca ranged from 1.04% in 4-year-old females to 3.58% in 4-year-old males).

The ability of an aquatic organism to absorb chemicals from the environment can be assessed by the BCF. Table 5 shows the values of BCF for each group of individuals. The accumulation coefficient was higher in the muscle than in the exoskeleton for Zn and Cu, and vice versa for Ca. BCF of Zn and Cu in the muscle and exoskeleton of spiny-cheek crayfish was much higher from water than from sediments, unlike Ca.

**Table 5:** Mean bioconcentration factor (BCF) of metals in the muscle and exoskeleton of 3- and 4-year-old females (♀) and males (♂) of spiny-cheek crayfish (*Faxonius limosus* Raf*.*) from sediments and water taken from Głowińsk reservoir (Poland)

	Individuals		Zn	Cu	Ca
Muscle/sediment	3 +	♀	3.44^a^	2.22	0.07^a^
		♂	4.04	2.85^a^	0.05^a^
	4 +	♀	4.76^b^	2.03	0.05^a^
		♂	4,29	2.05^b^	0.17^b^
Muscle/water	3 +	♀	5318^a^	2256	0.017^a^
		♂	6253	2895^a^	0.024^a^
	4 +	♀	7359^b^	2060	0.017^a^
		♂	6635	2087^b^	0.059^b^
Exoskeleton/sediment	3 +	♀	0,51^a^	0.21^a^	3.51
		♂	0,71^b^	0.44^b^	3.27
	4 +	♀	0,44^a^	0.21^a^	3.57
		♂	0,27^c^	0.18^a^	3.53
Exoskeleton/water	3 +	♀	795^a^	215^a^	1.23
		♂	1094^b^	445^b^	1.14
	4 +	♀	681^a^	215^a^	1.25
		♂	419^c^	179^a^	1.23
Exoskeleton/muscle	3 +	♀	0.15	0.10	50.61
		♂	0.17	0.15	68.94
	4 +	♀	0.09	0.10	74.24
		♂	0.06	0.09	20.98

Results of linear regression analysis with metal concentration as dependent variable and total length (TL) as independent variable (predictor) were presented in Table 6 (for muscle) and in Table 7 (for exoskeleton). Only in a few cases did the concentration of metals in the muscle significantly correlate with the total length (TL) of the crayfish. A statistically significant correlation (*P* < 0.05) between Zn concentration and TL in 3-year-old females and 4-year-old males and between Cu concentration and TL in 3-year-old males was observed (Table 6). The analyses of the coefficient of determination (*R*^2^), which informs us about the proportion of the variance for a dependent variable that is explained by an independent variable in a regression model, confirmed that the introduced predictor account for 55% to almost 90% of the total variation in its outcome variable (*R*^2^ = 0.5484, *R*^2^ = 0.7661, and *R*^2^ = 0.8989).

**Table 6:** Results of the significant linear models with metal concentration as response variable and total length (Tl) as predictor for the muscle of spiny-cheek crayfish (*Faxonius limosus* Raf.) taken from Głowińsk reservoir (Poland)

Muscle			Estimate	Standard error	***t***	***p***	***F***	***R***^**2**^	***y* = *ax* + *b***
3 + ♀	Zn	Intercept	0.1458	2.1176	11.066	0.000379	*F*_1.4_ = 35.59	0.8989	*y* = − 1.23*x* + 195.90
		TL	− 0.9989		− 5.966	0.003963			
3 + ♂	Zn	Intercept	− 0.6476	13.01	1.194	0.266724	*F*_1.8_ = 0.089	0.0110	*y* = 0.24*x* + 85.07
		TL	0.7539		0.299	0.772863			
4 + ♀	Zn	Intercept	− 0.8895	31.6596	− 0.796	0.470400	*F*_1.4_ = 1.29	0.2434	*y* = 4.17*x* – 295.34
		TL	0.9866		1.134	0.319990			
4 + ♂	Zn	Intercept	− 0.0751	8.9849	4.314	0.002566	*F*_1.8_ = 9.72	0.5484	*y* = − 2.95*x* + 406.17
		TL	− 0.9497		− 3.117	0.014285			
3 + ♀	Cu	Intercept	− 0.9720	5.3427	0.943	0.399005	*F*_1.4_ = 0.11	0.0277	*y* = − 0.18*x* + 42.13
		TL	0.9460		− 0.337	0.752870			
3 + ♂	Cu	Intercept	− 0.4452	4.9211	6.400	0.000209	*F*_1.8_ = 26.21	0.7661	*y* = − 1.56*x* + 172.49
		TL	− 0.9772		− 5.119	0.000908			
4 + ♀	Cu	Intercept	− 0.9717	5.2866	0.724	0.509132	*F*_1.4_ = 0.11	0.0257	*y* = − 0.20*x* + 44.84
		TL	0.9466		− 0.324	0.761477			
4 + ♂	Cu	Intercept	− 0.8017	6.9319	0.991	0.350631	*F*_1.8_ = 0.42	0.0497	*y* = − 0.47*x* + 71.99
		TL	0.5716		− 0.647	0.535958			
3 + ♀	Ca	Intercept	− 0.8660	1.6431	− 1.132	0.320597	*F*_1.4_ = 1.89	0.3216	*y* = 0.22*x* – 15.56
		TL	0.9891		1.377	0.240507			
3 + ♂	Ca	Intercept	− 0.6627	0.8254	0.281	0.786099	*F*_1.8_ = 0.05	0.0062	*y* = 0.01*x* + 1.27
		TL	0.7423		0.224	0.828719			
4 + ♀	Ca	Intercept	− 0.9704	0.3062	0.922	0.408928	*F*_1.4_ = 0.08	0.0188	*y* = − 0.01*x* + 3.31
		TL	0.9490		− 0.277	0.795190			
4 + ♂	Ca	Intercept	− 0.8491	2.2696	1.429	0.190879	*F*_1.8_ = 1.19	0.1293	*y* = − 0.26*x* + 33.98
		TL	0.4622		− 1.090	0.307412

**Table 7:** Results of the significant linear models with metal concentration as response variable and total length (Tl) as predictor for the exoskeleton of spiny-cheek crayfish (*Faxonius limosus* Raf.) taken from Głowińsk reservoir (Poland)

Exoskeleton			Estimate	Standard error	***t***	***p***	***F***	***R***^**2**^	***y* = *ax* + *b***
3 + ♀	Zn	Intercept	− 0.9647	2.1789	0.842	0.447089	*F*_1.4_ = 0.01	0.0025	*y* = − 0.02*x* + 15.34
		TL	0.9571		− 0.100	0.925133			
3 + ♂	Zn	Intercept	− 0.9695	0.8498	1.254	0.051248	*F*_1.4_ = 0.06	0.0151	*y* = − 0.02*x* + 20.13
		TL	0.9505		− 0.248	0.816547			
4 + ♀	Zn	Intercept	− 0.9064	1.8299	− 0.690	0.528006	*F*_1.4_ = 0.88	0.1800	*y* = 0.27*x* – 19.94
		TL	0.9841		0.937	0.401758			
4 + ♂	Zn	Intercept	− 0.9685	1.5673	0.703	0.520901	*F*_1.4_ = 0.05	0.0114	*y* = − 0.05*x* + 16.67
		TL	0.9520		− 0.215	0.840417			
3 + ♀	Cu	Intercept	− 0.8467	1.0891	− 1.272	0.272389	*F*_1.4_ = 2.42	0.3773	*y* = 0.16*x* – 11.58
		TL	0.9906		1.557	0.194519			
3 + ♂	Cu	Intercept	− 0.9726	2.2970	0.676	0.536246	*F*_1.4_ = 0.14	0.0308	*y* = − 0.07*x* + 11.30
		TL	0.9450		− 0.357	0.739265			
4 + ♀	Cu	Intercept	− 0.9891	1.3769	1.494	0.209361	*F*_1.4_ = 1.89	0.3214	*y* = − 0.29*x* + 32.48
		TL	0.8660		− 1.376	0.240706			
4 + ♂	Cu	Intercept	− 0.9354	0.6596	− 0.310	0.772037	*F*_1.4_ = 0.28	0.0645	*y* = 0.05*x* – 3.10
		TL	0.9767		0.525	0.627110			
3 + ♀	Ca	Intercept	− 0.8635	5.9418	1.994	0.116925	*F*_1.4_ = 1.96	0.3291	*y* = 0.81*x* + 99.05
		TL	0.9893		1.401	0.233931			
3 + ♂	Ca	Intercept	− 0.8459	12.4174	0.178	0.867161	*F*_1.4_ = 2.44	0.3793	*y* = 1.63*x* + 16.11
		TL	0.9906		1.563	0.192996			
4 + ♀	Ca	Intercept	− 0.9858	6.3200	1.781	0.059778	*F*_1.4_ = 1.13	0.2199	*y* = − 1.04*x* + 277.41
		TL	0.8959		− 1.062	0.348065			
4 + ♂	Ca	Intercept	− 0.9284	9.0050	0.606	0.576961	*F*_1.4_ = 0.41	0.0923	*y* = 0.88*x* + 82.66
		TL	0.9790		0.638	0.558420			

Table 8 presents the content of Ca, Cu, and Zn determined in the muscle of crayfish calculated for 100 g of wet weight and % of the RDI for the analysed minerals. The research indicated that crayfish muscle was a rich source of Cu (over 100% RDI), and consumption of 100 g of spiny-cheek crayfish muscle could meet the daily requirement for Zn up to 27.5% and for Ca in 12.4%.

**Table 8:** Metal concentration in the analysed muscle of spiny-cheek crayfish (*Faxonius limosus* Raf*.*), recommended daily intake (RDI) of metals, and % RDI for 100 g of spiny-cheek crayfish (*Faxonius limosus* Raf.) muscle taken from Głowińsk reservoir (Poland)

Metals	RDI^*^ (mg)	Content in the crayfish muscle (mg 100 g^−1^ w.w.)	%RDI for 100 g of muscle
Ca	700–1300	86.87	6.68–12.41%
Zn	8–13	2.20	16.92–27.50%
Cu	0.3–1.3	0.56	43.08– > 100%

^*^RDI, recommended daily intake according to nutrition standards developed by the National Food and Nutrition Institute in Warsaw (Jarosz et al. 2020)

## Discussion

Supporting previous literature, Ca accumulated in the highest amounts in the exoskeleton, while Zn and Cu in the muscle (Nędzarek et al. 2020; Śmietana et al. 2020). Our research confirmed that concentration of metals in the abdominal muscle and the exoskeleton of spiny-cheek crayfish was in this decreasing order Ca > Zn > Cu. The same results for muscle were observed by Protasowicki et al. (2013) in signal crayfish (*Pacifastacus leniusculus* [Dana 1852]) from Mazurian Lakes, by Goretti et al. (2016) in red swamp crayfish (*Procambarus clarkii* [Girard 1852]) from Lake Trasimeno, and by Varol and Sünbül (2018) in mud crayfish (*Astacus leptodactylus* [Eschscholz 1823]) from the Keban Dam Reservoir in Turkey. A higher level of Cu than Zn in exoskeleton was observed by Nędzarek et al. (2020) in signal crayfish exoskeleton from the Wieprza River and by Mistri et al. (2020) in red swamp crayfish of the Po River Delta area.

Calcium (Ca) is one of the macroelements responsible for building the skeletal system and maintaining a proper acid–base balance, and as confirmed by the research of Lall (2002), muscle tissue is not the primary site of Ca accumulation, unlike the scales, bones, and skin. This was confirmed by our analyses of a spiny-cheek crayfish, which showed calcium concentrations about 40 times higher in the exoskeleton compared to the muscle tissue (Table 3). This is due to chitin, which builds the crustacean exoskeleton, which is made of calcium carbonate and has a high sorption capacity for metals, which is due to passive adsorption of metals from water (Mistri et al. 2020). Hence, this tissue may be a rich source of Ca and can be used as a natural fertiliser for plants. Nędzarek et al. (2020) recorded 260 to 500 times higher Ca level in the exoskeleton compared to the muscle for the signal crayfish. Our research did not confirm significant differences in the level of Ca in the exoskeleton between groups. Neither sex nor age was a significant factor affecting the level of Ca in this tissue. As Wærvågen et al. (2016) confirmed, moult timing and frequency, as well as length increment per moult, have a major impact on exoskeleton calcification in Norwegian populations of the noble crayfish.

Zn is a microelement that plays an important role in the proper functioning of an organism. This metal is responsible for carbohydrates, proteins, nucleic acid metabolism, proper bone mineralization, and the functioning of the immune system (Chavez-Sanchez et al. 2000). Our analyses of spiny-cheek crayfish demonstrated about 8.5 times higher concentration of Zn in muscle compared to the exoskeleton (Table 3). Nędzarek et al. (2020) found about 3 times higher Zn levels in signal crayfish muscle compared to the exoskeleton. The same results were denoted by Mistri et al. (2020) in red swamp crayfish (the ratio was from 3.5 to 4.8 times). The studies of Jaafarzadeh et al. (2015) confirmed that the chitin extracted from the shrimp was an effective Zn adsorbent, but the contact time or individual predispositions strongly influenced the efficiency of biosorption.

A microelement that plays a significant role in the production of red blood cells, synthesis of nucleic acids, hardening of collagen and keratinization of hair, haemocyanin biosynthesis, and catabolism in aquatic arthropods and molluscs is copper (Cu) (Mistri et al. 2020). Our research showed almost 9 times higher concentration of this metal in muscle compared to the exoskeleton (Table 3). The highest concentration of this metal in the exoskeleton was found in 3-year-old males of spiny-cheek crayfish. Nędzarek et al. (2020) found about 1.3 times higher Cu levels in signal crayfish muscle compared to the exoskeleton. The same results were denoted by Mistri et al. (2020) in red swamp crayfish (the ratio was from 1.3 to 3.4 times). The studies of Soedarini et al. (2012) on red swamp crayfish showed that the muscle tissue was characterised by the lowest degree of Cu absorption compared to other tissues, which confirms a greater degree of elimination than accumulation. No significant differences in Cu concentration in the abdominal muscle between females and males of spiny-cheek crayfish within one age group were observed. The only significant differences were found in the Cu concentration in muscle between 3- and 4-year-old males, which were in line with Balzani et al. (2022). These differences are likely due to the ability of crayfish to clear metals rapidly, and this is why these animals are useful for assessing Cu bioavailability in aquatic ecosystems in a short-term monitoring programme (Kouba et al. 2010; Xiong et al. 2020). As demonstrated by Mistri et al. (2020), the large accumulation of Cu in the abdominal muscle may also be caused by a physiological response to external stress.

Statistically significant differences in the content of the determined metals between 3- and 4-year-old crayfish were determined for Zn, Cu and Ca in the muscle and for Cu and Zn in the exoskeleton. The analyses showed significant differences in the concentration of Zn in muscle between 3- and 4-year-old females and in the concentration of Cu and Ca between 3- and 4-year-old males. As confirmed by Jaafarzadeh et al. (2015) in studies performed on shrimp shells, the period of exposure to the environmental factor is responsible for the level of accumulation of metals such as Zn. This is consistent with our previous research, which confirmed the significant influence of the age of spiny-cheek crayfish on the Zn and Cu levels in muscle (Stanek et al. 2017). Lower metal values in the muscle of older individuals (for example, Cu in 4-year-old spiny-cheek crayfish) may be due to a dilution effect (Balzani et al. 2022). When the metabolic rate is faster, as is often the case in younger organisms, then the growth dilution effect will be greater (Evans-Illidge 1997).

Statistically significant differences in the content of the determined metals between males and females were determined for Ca in the muscle and for Zn in the exoskeleton. Hagen and Sneddon (2009) and Nędzarek et al. (2020) showed no significant difference between males and females crayfish in Zn and Cu content in the muscle. Naghshbandi et al. (2007) confirmed differences in Zn and Cu content in the muscle between sexes for mud crayfish. Chen et al. (2005) confirmed the lack of significant differences in Cu concentration between individuals of different sexes in any tissue. One hypothesis is that Cu is an essential element for the blood pigments of crustaceans, and its requirement should not differ between the sexes. Differences in the concentration of this metal may appear in small crustaceans or juveniles with little or no haemocyanin, which may result in an increase in the concentration of Cu in the body as the size of the animal increases. Kaya et al. (2015) confirmed by scanning electron microscopy (SEM) that the male chitin surface structure contained 25–90 nm wide nanofibres and 90–250 nm nanopores, while no pores or nanofibres were observed in the chitin surface structure of the majority of females (nanofibres were observed only in *Melanogryllus desertus* females). Moreover, they confirmed the differences in dry matter of chitin between species (these values ranged from 4.71 to 11.84%), and they observed that the quantity of chitin was greater in males than in females. The research of Jussila et al. (1995) on noble crayfish confirmed differences in the metal content of the exoskeleton between males and females may be due to the difference in the frequency of moulting during the year. This was confirmed by the research of Nędzarek et al. (2020) on the level of metals in the exoskeleton of signal crayfish from the Wieprza River. Differences in the metal content of the muscle between males and females may be due to differing growth rates between sexes (Evans-Illidge 1997). Moreover, research concerning *Nephrops norvegicus* shows that males have higher feeding rates than females, and the concentration of metals may be higher (Canli and Furness 1993).

According to Tao et al. (2012), a BCF value > 1 means that the organism has the potential to accumulate a chemical substance, and BCF value > 100 means high accumulation capacity. Other literature sources indicate that a BCF value of < 1000 means that the chemical is not significantly bioaccumulative, with BCF in the range of 1000–5000, the chemical substance has a potential to bioaccumulate, and with BCF > 5000, the chemical substance shows high bioaccumulation (Costanza et al. 2012; Varol and Sünbül 2018). The calculated BCF values for Zn were consistent with the data obtained by Nędzarek et al. (2020) for signal crayfish, and BCF values for Cu were the same as those obtained by Varol and Sünbül (2018) for mud crayfish. Our analyses confirmed that Zn and Cu accumulate mainly in the muscle and Ca in the exoskeleton. This is because Ca is the main constituent of the exoskeleton as calcium carbonate (30–40%), in addition to chitin (20–30%) and protein (20–30%) (Chen et al. 2020). The analyses of spiny-cheek crayfish showed a much higher bioaccumulation capacity of Zn and Cu in the muscle from water (BCF > 1000) and a greater bioaccumulation capacity of Ca in the exoskeleton from sediments (BCF > 3). Since the exoskeleton is in direct contact with the environment, metals accumulate in this tissue through passive adsorption from water or sediment rather than bioaccumulation (Mistri et al. 2020). The differences in the bioaccumulation of metals in tissue by elimination compared to other tissues can be expressed as the ratio of the levels found in these tissues (for example, ratio exoskeleton/muscle). BCF values for Zn and Cu were similar and were below 1, while in the case of Ca, BCF value was in the range of 20.98 to 74.24. Two factors influence the BCF values in different tissues: different biochemical functions of elements and different physiological functions of individual organs (Nędzarek et al. 2020). In order to confirm this tendency of metal accumulation in the analysed crayfish species, studies on individuals obtained from reservoirs with various degrees of eutrophication should be carried out.

The lack of significant relationships between total length and the concentration of metals in the muscle may result from the dilution of metals as the animal's body grows, moreover, it may indicate faster metabolism in younger animals compared to older ones (Balzani et al. 2022), and it may also result from the growth-hindering effect of metals. Conversely, a positive correlation indicates a trend of metal accumulation as the animal grows (Ergen et al. 2015). Significant negative correlations between the concentration of metals and TL in the exoskeleton may result from cyclic moulting, which results in the removal of metals. Our analyses on spiny-cheek crayfish supported this. The degree of Zn accumulation in crayfish tissues depends on the concentration of Ca in the water and the correlation between these metals, and it is largely due to surface adsorption on the shell and gills (Bryan 1967). The hypothesis is that Ca has the propensity to form complexes with phytate and Zn that are insoluble and consequently have an inhibitory effect on Zn absorption (Lönnerdal 2000). Our preliminary analyses confirmed the lack of significant correlations between the concentration of Zn and Ca in each of the studied groups.

The results of the RDI analysis for spiny-cheek crayfish were in line with Nędzarek et al. (2020), who showed that the consumption of a portion of 100 g of signal crayfish muscle from the Wieprza River meets 3 to 10% of the dietary reference intake (DRI) for K, Ca, and Fe. In the case of Zn DRI, it was lower for muscle from abdomen of males and females (about 23%) and much higher for muscle with their claws (over 60%). Only in the case of Cu was DRI exceeded, with the range from 88 to 164% of DRI. As confirmed by Śmietana et al. (2021), the consumption of 100 g of chelae muscle of spiny-cheek crayfish from Lake Sominko covers 103.7 and 54.8% of the consumer’s daily demand for Zn and Ca, respectively. The consumption of 100 g of abdomen muscle mostly covers the demand for Zn (17.08%). Moreover, according to the data of the US Food and Drugs Administration, the maximum allowable level is 6.1 mg kg^−1^ dw for Cu and 21.2 mg kg^−1^ dw for Zn (Alcorlo et al. 2006), which makes the analysed spiny-cheek crayfish suitable for human consumption.

## Conclusions

Factors influencing the differences in the accumulation of metals in crayfish tissues are age, which is equivalent to the time of exposure of the animal to metals, and sex, which determines the moulting cycle, different physiological conditions, and elimination processes. This hypothesis was supported by spiny-cheek crayfish analyses. For most metals, significant differences were found between 3- and 4-year-old individuals both in the muscle and exoskeleton. The analyses of spiny-cheek crayfish partially supported the hypothesis of the BCF, as this mechanism depends on the type of metal. Knowledge of the mechanisms of metal bioaccumulation and the factors influencing it is an important contribution to the environmental monitoring of freshwater ecosystems. As evidenced by the studies of other authors, environmental conditions are very important, especially pH (which affects the degree of mineralization); therefore, this mechanism requires further research. Analyses of the RDI for the investigated minerals and data from the US Food and Drugs Administration, the maximum allowable level of Zn and Cu confirmed that consumption of crayfish meat would be safe for the health of potential consumers in terms of Ca, Zn, and Cu levels.

## Data Availability

The datasets generated during and/or analysed during the current study are available from the corresponding author upon reasonable request.

## Materials availability

The datasets generated during and/or analysed during the current study are available from the corresponding author upon reasonable request.
